# Low-intensity pulsed ultrasound promotes proliferation and migration of HaCaT keratinocytes through the PI3K/AKT and JNK pathways

**DOI:** 10.1590/1414-431X20187862

**Published:** 2018-10-18

**Authors:** Xiaoyan Leng, Jing Shang, Danhui Gao, Jiang Wu

**Affiliations:** 1 Department of Ultrasound, Chengyang People's Hospital, Qingdao, China; 2 Health Management Center, The Affiliated Hospital of Qingdao University, Qingdao, China; 3 Department of Vascular Surgery, The Affiliated Hospital of Qingdao University, Qingdao, China

**Keywords:** Low-intensity pulsed ultrasound, HaCaT cells, Proliferation, Migration, PI3K/AKT/JNK

## Abstract

Although the effects of low-intensity pulsed ultrasound (LIPUS) on diverse cell types have been fully studied, the functional role of LIPUS in keratinocytes remains poorly understood. This study aimed to investigate the effects of LIPUS on proliferation and migration of HaCaT cells as well as the regulatory mechanisms associated with signaling pathways. Human HaCaT cells were exposed or not to LIPUS, and cell proliferation and migration were measured by BrdU incorporation assay and Transwell assay, respectively. Expression of proteins associated with proliferation and migration was evaluated by western blot analysis. Expression of key kinases in the PI3K/AKT and JNK pathways was also evaluated by western blot analysis. Effects of LIPUS on the PI3K/AKT and JNK pathways, and whether LIPUS affected HaCaT cells via these two pathways were finally explored. When the parameter of LIPUS (number of cycles) was set at 300, cell viability was the highest after LIPUS stimulation. We then found that the percentage of BrdU positive cells was enhanced by LIPUS, along with up-regulation of cyclinD1, CDK6, CDK4, and VEGF. LIPUS promoted migration, as well as up-regulation of MMP-2 and MMP-9. Phosphorylation levels of key kinases in the PI3K/AKT and JNK pathways were increased by LIPUS. Inhibition of either PI3K/AKT pathway or JNK pathway attenuated effects of LIPUS on HaCaT cells, and co-inhibition of these two pathways showed augmented effects. LIPUS promoted proliferation and migration of HaCaT cells through activating the PI3K/AKT and JNK pathways.

## Introduction

Low-intensity pulsed ultrasound (LIPUS) is defined as a non-invasive ultrasound technique with low frequency and low intensity (1,2). Several studies have reported the effective influence of LIPUS on fracture healing. One study has shown that LIPUS can promote fracture healing through accelerating callus formation, angiogenesis, and callus remodeling (3). Another study has proven that LIPUS stimulation increases migration of mesenchymal stem cells (MSCs) towards the fracture site and thereby improves fracture healing (4). The functional role of LIPUS in osteogenic differentiation and the maturation of osteoblasts is also an explanation for the role of LIPUS in fracture healing (5). In addition to fracture healing, pleiotropic and complex bio-effects of LIPUS have been identified recently. Lin et al. (6) have reported that LIPUS protects rats against aluminum-induced cerebral damage in Alzheimer's disease rat model. Left ventricular dysfunction in mice with acute myocardial infarction could be attenuated by LIPUS (7). Biological effects of reagents and drugs were augmented by LIPUS, such as 5-aminolevulinic acid (8).

Skin, comprised of keratinocytes, melanocytes, and fibroblasts, is the largest organ of the human body, and it provides a protective barrier between the internal milieu and the environment (9,10). Keratinocytes are the main cells in the epidermis, which are closely associated with skin diseases such as psoriasis and atopic dermatitis as well as skin wound healing (11–13). Previous studies have demonstrated that LIPUS generates biochemical events at the cellular level (14,15). Therefore, the effects of LIPUS stimulation on different cell types may indicate innovative therapeutic effects of LIPUS. As keratinocytes are the first response cells to external stimuli, we hypothesized that there might be some changes in keratinocytes after LIPUS stimulation, which might reveal novel applications of LIPUS as treatment for diseases related to keratinocytes. However, to our knowledge, the related literature is limited.

HaCaT cells, human spontaneously immortalized keratinocytes with full epidermal differentiation capacity, have been widely used as an *in vitro* keratinocyte model (9,16). In this study, we explored the effects of LIPUS on HaCaT keratinocytes. Since the proliferative and migratory potentials of keratinocytes are two important aspects for diverse diseases related to these cells, the effects of LIPUS on proliferation and migration of HaCaT cells were investigated. Moreover, the regulatory mechanisms of LIPUS in HaCaT cells associated with signaling pathways were preliminarily studied.

## Material and Methods

### Cell culture and treatment

Human epidermal keratinocyte cell line HaCaT was obtained from CLS Cell Lines Service (Germany). HaCaT cells were cultured in Dulbecco's modified Eagle's medium (DMEM; Gibco, USA) supplemented with 10% fetal bovine serum (FBS; Gibco). Cell maintenance was performed in a humidified incubator at 37°C with 5% CO_2_ and 95% air. For inhibition of the PI3K/AKT pathway or the JNK pathway, cells were incubated in DMEM containing LY294002 (10–50 μM, Sigma-Aldrich, USA) or SP600125 (1–10 μM, Sigma-Aldrich) for 1 h prior to LIPUS stimulation.

### LIPUS stimulation

The LIPUS exposure device consists of a signal generator (Agilent Technologies, USA), wideband power amplifier (Electronics and Innovation Ltd., USA), and a planar transducer (Chongqing Haifu Medical Technology Co., Ltd., China). When cells reached confluence, culture dishes were plated on the transducer (diameter 6 cm), which was filled with degassed water. For LIPUS stimulation, the frequency of planar transducer was set at 0.5 MHz, voltage was set at 150 MVpp, the number of cycles was set at 100, 200, 300, 400, or 500, and the spatial temporal average sound pressure was set at 0.3 MPa. Cells in the LIPUS group were exposed to LIPUS stimuli for 1 min, whereas cells in the control group were treated identically without LIPUS stimuli. The temperature of the cell culture was kept at 37°C.

### Cell viability assay

Viability of HaCaT cells was measured by using the Cell Counting Kit-8 (Dojindo, Japan). Briefly, cells exposed or not to LIPUS were seeded into 96-well plates at 5×10^3^ cells per well, and then cells were maintained at 37°C for 48 h. Subsequently, 10 μL of CCK-8 solution was added into the culture medium, followed by incubation at 37°C for 1 h. Absorbance at 450 nm was detected using a Microplate Reader (Bio-Rad, USA).

### Proliferation assay

Proliferation of HaCaT cells was analyzed using bromodeoxyuridine (BrdU) incorporation assay. Briefly, cells exposed or not to LIPUS were seeded into 96-well plates at 2×10^3^ cells per well, and cells were maintained at 37°C for 48 h. Then, 20 μL BrdU from the BrdU Cell Proliferation ELISA Kit (Abcam, UK) was added into the culture medium, followed by incubation at 37°C for 3 h. After incubation with anti-BrdU antibody and peroxidase-conjugated goat anti-mouse IgG, successively, 100 μL of TMB peroxidase substrate was added and the mixture was kept at room temperature for 30 min in the dark. The reaction was stopped using Stop Solution, and absorbance at a dual wavelength of 450/550 nm was measured by a Microplate Reader.

### Migration assay

Migration of HaCaT cells was tested using 24-well plates with Falcon cell culture inserts (8-μm pores; Corning, USA). Briefly, cells exposed or not to LIPUS were suspended in 200 μL DMEM and then added into the upper chamber. DMEM containing 10% FBS (600 μL) was added into the lower chamber. Cells were incubated at 37°C for 48 h, followed by cell fixation using methanol. The non-migratory cells on the upper surface of the inserts were removed carefully, and the cells on the lower side of the inserts were stained with 0.1% crystal violet. Migratory cells were counted under a microscope (Olympus, Japan) in five randomly selected fields.

### Western blot analysis

After exposure or not to LIPUS, HaCaT cells were lysed in RIPA buffer (Beyotime, China) containing 1 mM phenylmethyl sulfonyl fluoride (PMSF; Beyotime). The whole lysates were centrifuged at 12,000 *g* for 10 min at 4°C, and the protein levels in the supernatants were quantified using the BCA™ Protein Assay Kit (Pierce, USA). Proteins were loaded onto SDS-PAGE gels and electroblotted onto polyvinylidene difluoride (PVDF) membranes. Then, PVDF membranes were blocked with 5% bovine serum albumin (BSA; Solarbio, China), and incubated overnight at 4°C with specific primary antibodies for cyclin D1 (ab134175), cyclin-dependent kinase (CDK) 6 (ab151247), CDK4 (ab137675), vascular endothelial growth factor (VEGF; ab150766), matrix metalloproteinase (MMP) 2 (ab97779), MMP-9 (ab137867), PI3K (ab180967), phospho (p)-PI3K (ab182651), β-actin (ab8227, all Abcam), AKT (#9272), p-AKT (#9271), JNK (#9252), and p-JNK (#9251, all Cell Signaling Technology, USA). After rinsing, PVDF membranes were incubated with HRP-conjugated secondary antibody (goat anti-rabbit, ab205718, Abcam) at room temperature. An enhanced chemiluminescence (ECL) kit (Thermo Scientific, USA) was used to detect the target proteins. Protein levels were quantified by ImageJ software (National Institutes of Health, USA).

### Statistical analysis

Data are reported as means±SD of three independent experiments. Statistical analysis was performed using Graphpad Prism 5 software (USA). The P-values were determined by Student's *t*-test for comparison between two groups, and by one-way analysis of variance (ANOVA) with Bonferroni's correction for comparison among three or more groups. P<0.05 was considered as a significant difference.

## Results

### LIPUS promoted proliferation of HaCaT cells

**Figure 1.**
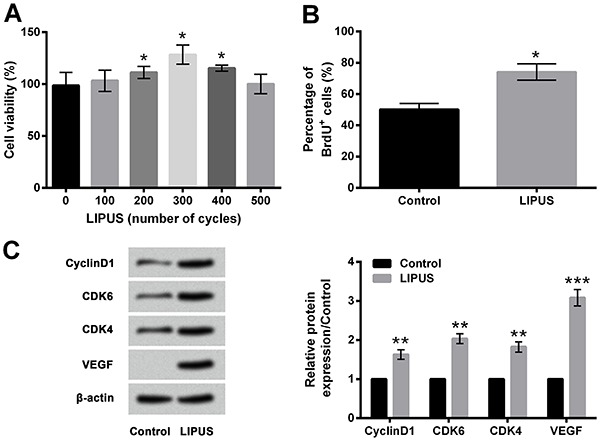
Low-intensity pulsed ultrasound (LIPUS) promoted proliferation of HaCaT cells. The parameter of LIPUS stimulation (number of cycles) was set at 100–500. *A*, HaCaT cell viability was determined by CCK-8 assay. *B*, Percentage of BrdU positive cells was evaluated by BrdU incorporation assay. *C*, Expression of proteins associated with proliferation was evaluated by western blot analysis. Data are reported as means±SD of three independent experiments. *P<0.05, **P<0.01, ***P<0.001 compared to control (Student's *t*-test).

After LIPUS stimulation, during which the number of cycles was set at 100–500, viability of HaCaT cells was measured. Results in Figure 1A show that cell viability was significantly enhanced when the number of cycles was 200, 300, and 400 compared to the unstimulated cells (all P<0.05). As the cell viability was the highest when the number of cycles was 300, the parameter of LIPUS (the number of cycles) was set at 300 in subsequent experiments. Then, we found that the percentage of BrdU positive cells in the LIPUS group was markedly higher than the control group (P<0.05, Figure 1B). Likewise, western blot analysis showed expression levels of cyclinD1, CDK6, CDK4, and VEGF were significantly up-regulated by LIPUS compared with the control group (P<0.01 or P<0.001, Figure 1C). Results illustrated that LIPUS stimulation could promote HaCaT cell proliferation.

### LIPUS promoted migration of HaCaT cells

**Figure 2.**
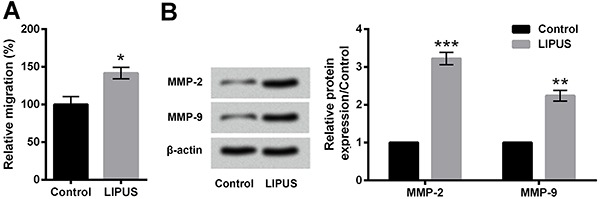
Low-intensity pulsed ultrasound (LIPUS) promoted migration of HaCaT cells. HaCaT cells were exposed to LIPUS, and unstimulated cells acted as control. *A*, Relative migration was determined by Transwell assay. *B*, Expression of proteins associated with migration was evaluated by western blot analysis. Data are reported as means±SD of three independent experiments. *P<0.05, **P<0.01, ***P<0.001 compared to control (Student's *t*-test).

After LIPUS stimulation, relative migration of HaCaT cells was analyzed. As shown in Figure 2A, relative migration of cells in the LIPUS group was significantly higher than the control group (P<0.05). The expression of proteins associated with cell migration was also evaluated. Expression levels of MMP-2 and MMP-9 were significantly enhanced by LIPUS stimulation compared with the control group (P<0.01 or P<0.001, Figure 2B). Results illustrated that LIPUS stimulation could promote HaCaT cell migration.

### LIPUS activated the PI3K/AKT and JNK pathways in HaCaT cells

**Figure 3.**
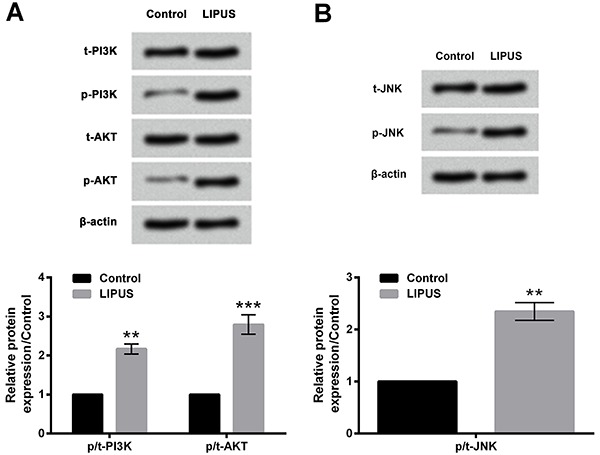
Low-intensity pulsed ultrasound (LIPUS) activated the PI3K/AKT and JNK pathways in HaCaT cells. Expression of key kinases in the PI3K/AKT pathway (*A*) and the JNK pathway (*B*) were evaluated by western blot. Data are reported as means±SD of three independent experiments. **P<0.01, ***P<0.001 compared to control (Student's *t*-test).

Effects of LIPUS on the signaling pathways were evaluated. After LIPUS stimulation, phosphorylation levels of PI3K and AKT were markedly increased relative to the control group (P<0.01 or P<0.001, Figure 3A). Similarly, alteration of p-JNK after LIPUS was consistent with p-PI3K and p-AKT, showing significantly elevated phosphorylation after LIPUS stimulation (P<0.01, Figure 3B). Results indicated that LIPUS could activate the PI3K/AKT and JNK pathways in HaCaT cells.

### LIPUS affected HaCaT cells via the PI3K/AKT and JNK pathways

**Figure 4.**
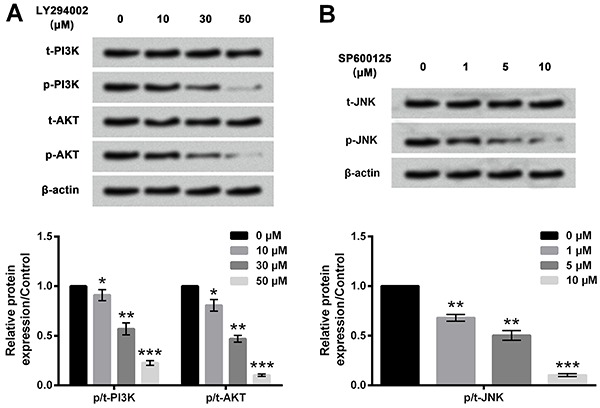
Stimulation with LY294002 (*A*) or SP600125 (*B*) for 1 h inhibited the PI3K/AKT pathway or JNK pathway in HaCaT cells, respectively. Expression of key kinases in the PI3K/AKT and JNK pathways were tested by western blot. Data are reported as means±SD of three independent experiments. *P<0.05, **P<0.01, ***P<0.001 compared to control (ANOVA).

**Figure 5.**
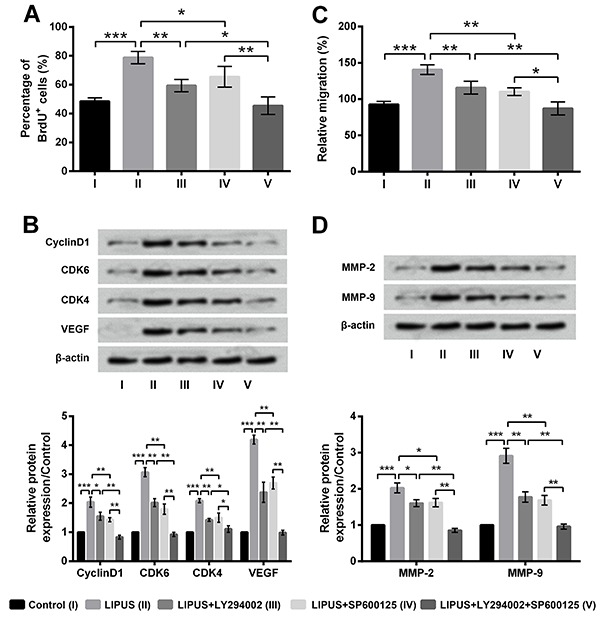
Low-intensity pulsed ultrasound (LIPUS) promoted proliferation and migration of HaCaT cells via the PI3K/AKT and JNK pathways. *A*, Percentage of BrdU positive cells was evaluated by BrdU incorporation assay. *B*, Expression of proteins associated with proliferation was evaluated by western blot. *C*, Relative migration was determined by the Transwell assay. *D*, Expression of proteins associated with migration was evaluated by western blot. Data are reported as means±SD of three independent experiments. *P<0.05, **P<0.01, ***P<0.001 (ANOVA).

We verified whether activation of the PI3K/AKT and JNK pathways was the reason for the effects of LIPUS on HaCaT cells. With the increase of LY294002 concentration, phosphorylation levels of PI3K and AKT were notably reduced compared with the untreated cells (P<0.05, P<0.01 or P<0.001, Figure 4A). Similarly, phosphorylation levels of JNK were significantly reduced with the increase of SP600125 concentration (P<0.01 or P<0.001, Figure 4B). Results suggested that stimulation with LY294002 and SP600125 could inactivate the PI3K/AKT pathway and the JNK pathway, respectively. Then, HaCaT cells were pre-treated with LY294002 and/or SP600125, followed by LIPUS stimulation. Proliferation and migration of HaCaT cells were measured. Percentage of BrdU positive cells as well as expression levels of cyclinD1, CDK6, CDK4, and VEGF was significantly reduced by either LY294002 or SP600125 relative to the LIPUS group (P<0.05 or P<0.01, Figure 5A and B). Meanwhile, relative migration as well as expression levels of MMP-2 and MMP-9 was decreased by either LY294002 or SP600125 relative to the LIPUS group (P<0.05 or P<0.01, Figure 5C and D). Moreover, the effects of pretreatment with LY294002 and SP600125 on HaCaT cells were more powerful than pretreatment with LY294002 or SP600125 (P<0.05 or P<0.01). Results collectively illustrated that LIPUS might affect HaCaT cells by activating the PI3K/AKT and JNK pathways.

## Discussion

Although the effects of LIPUS stimulation on MSCs, visceral pre-adipocytes, and cardiomyocytes have been reported previously, the functional role of LIPUS in HaCaT cells remains poorly understood. In this study, we reported for the first time that LIPUS promoted proliferation and migration of HaCaT cells, along with up-regulation of cyclinD1, CDK6, CDK4, VEGF, MMP-2, and MMP-9. The PI3K/AKT and JNK pathways were both significantly activated after LIPUS stimulation, and inhibition of these two pathways showed reversed effects on proliferation and migration in LIPUS-treated cells.

Effects of LIPUS on proliferation were significantly distinct in different cell types. Xu et al. (2) have demonstrated that LIPUS represses proliferation in rat visceral pre-adipocytes. Conversely, Ling et al. (17) have proven that proliferation of human amnion-derived MSCs is promoted by LIPUS. Herein, effects of LIPUS on the proliferation of HaCaT cells were studied. BrdU is a thymidine analog, which can mark cells undergoing division, thus BrdU is widely used as a marker of cell proliferation (18). CyclinD1 is a cell cycle regulatory protein, and its overexpression suggests uncontrolled cell proliferation (19). CyclinD1 can activate CDK4 and CDK6 and then causes phosphorylation of protein substrates involved in cell cycle progression, resulting in elevated proliferation (20). VEGF is a powerful inducer of angiogenesis and can stimulate growth and proliferation of HaCaT cells (21,22). In our study, the increase of BrdU positive cells and up-regulation of cyclinD1, CDK4, CDK6, and VEGF induced by LIPUS suggested that LIPUS could promote HaCaT cell proliferation. The pro-proliferative role of LIPUS in HaCaT cells was consistent with the beneficial effects of LIPUS on proliferation of fibroblasts, osteoblasts, and chondrocytes, which were well documented by Aliabouzar et al. (23). The up-regulation of VEGF by LIPUS in HaCaT cells was consistent with that in parietal cortex cells (24) and MSCs (25).

Like proliferation, effects of LIPUS on migration were also reported to be distinct in different cell types. For example, LIPUS promotes migration of periodontal ligament stem cells (26), osteoblasts (27), and chondrogenic progenitor cells (28). However, cell migration of human aortic endothelial cells was suppressed by LIPUS (29). Herein, the effects of LIPUS on HaCaT cell migration were subsequently studied. MMPs are proteases that can degrade extracellular matrix and thereby lead to cell migration. Among diverse MMPs, MMP-2 and MMP-9 are the most crucial and many studies have found that elevated expression of MMP-2 and MMP-9 is accompanied by promoted migration (30,31). Transwell results in our study suggested that LIPUS could promote HaCaT cell migration, and the up-regulation of MMP-2 and MMP-9 after LIPUS stimulation supported the conclusion. The pro-migratory role of LIPUS in HaCaT cells was consistent with that in periodontal ligament stem cells, osteoblasts and chondrogenic progenitor cells described above.

Several signaling pathways are involved in the modulation of LIPUS. The PI3K/AKT and JNK pathways are two important signaling pathways related to proliferation and migration (32). It has been reported that LIPUS exerts a pro-proliferative role in amnion-derived MSCs through activation of the PI3K/AKT pathway (17). The JNK pathway in MSCs was activated by LIPUS, along with elevated proliferation (33). Formononetin was reported to repress migration by down-regulating MMP-2 and MM-9 via activation of the PI3K/AKT pathway (34). Up-regulation of MMP-2 and MMP-9 induced by nicotine in RAW264.7 and MOVAS cells was accompanied by activation of the JNK pathway (35). Therefore, we hypothesized that the PI3K/AKT and JNK pathways might be involved in LIPUS-related modulations. First, we proved that LIPUS stimulation activated the PI3K/AKT and JNK pathways in HaCaT cells. Then, we used LY294002 and SP600125 to inhibit the PI3K/AKT and JNK pathways, respectively. Finally, results showed that the effects of LIPUS on HaCaT cells were mitigated by either inhibition of the PI3K/AKT pathway or inhibition of the JNK pathway. Moreover, the effects were further mitigated by co-inhibition of the PI3K/AKT and JNK pathways.

Among the pathophysiological processes associated with keratinocytes, wound healing is a complex process involving the proliferation and migration of unwounded keratinocytes nearby (36). Increasing proliferation and migration of keratinocytes have been reported to promote skin wound healing (13). Therefore, the pro-proliferative and pro-migratory role of LIPUS in HaCaT cells suggested a potential application of LIPUS in wound healing therapy, which needs more experiments performed in animals to support the hypothesis.

In conclusion, our study reported for the first time that LIPUS promoted proliferation and migration of HaCaT cells through activation of the PI3K/AKT and JNK pathways. This study expanded the potential application of LIPUS in treatment of skin lesion-related diseases.
